# A Dynamic Neuro-Fuzzy Model Providing Bio-State Estimation and Prognosis Prediction for Wearable Intelligent Assistants

**DOI:** 10.1186/1743-0003-2-15

**Published:** 2005-06-28

**Authors:** Yu Wang, Jack M Winters

**Affiliations:** 1 Department of Biomedical Engineering, Marquette University, Milwaukee, WI, USA

## Abstract

**Background:**

Intelligent management of wearable applications in rehabilitation requires an understanding of the current context, which is constantly changing over the rehabilitation process because of changes in the person's status and environment. This paper presents a dynamic recurrent neuro-fuzzy system that implements expert-and evidence-based reasoning. It is intended to provide context-awareness for wearable intelligent agents/assistants (WIAs).

**Methods:**

The model structure includes the following types of signals: inputs, states, outputs and outcomes. Inputs are facts or events which have effects on patients' physiological and rehabilitative states; different classes of inputs (e.g., facts, context, medication, therapy) have different nonlinear mappings to a fuzzy "effect." States are dimensionless linguistic fuzzy variables that change based on causal rules, as implemented by a fuzzy inference system (FIS). The FIS, with rules based on expertise and evidence, essentially defines the nonlinear state equations that are implemented by nuclei of dynamic neurons. Outputs, a function of weighing of states and effective inputs using conventional or fuzzy mapping, can perform actions, predict performance, or assist with decision-making. Outcomes are scalars to be extremized that are a function of outputs and states.

**Results:**

The first example demonstrates setup and use for a large-scale stroke neurorehabilitation application (with 16 inputs, 12 states, 5 outputs and 3 outcomes), showing how this modelling tool can successfully capture causal dynamic change in context-relevant states (e.g., impairments, pain) as a function of input event patterns (e.g., medications). The second example demonstrates use of scientific evidence to develop rule-based dynamic models, here for predicting changes in muscle strength with short-term fatigue and long-term strength-training.

**Conclusion:**

A neuro-fuzzy modelling framework is developed for estimating rehabilitative change that can be applied in any field of rehabilitation if sufficient evidence and/or expert knowledge are available. It is intended to provide context-awareness of changing status through state estimation, which is critical information for WIA's to be effective.

## Background

Emerging wearable technologies are expected to constitute an important component of the vision of user-centered, 21^st^-century rehabilitative healthcare [1-4]. Indeed, the consensus recommendations of a workshop on future homecare technologies envisioned intelligent wearable sensors as one of the top trends [1]. The top two knowledge gaps that were identified targeted the need for better [1,2]:

1. information reduction algorithms and sense-making tools, and

2. outcomes and functional assessment tools.

This project addresses these gaps in knowledge for the area of rehabilitative healthcare.

The first of these recognizes the challenge of effectively integrating and using the massive amount of sensor-based data that can be potentially be collected. It is well established in the intelligent systems community that a key barrier to intelligent use of information is context-awareness. With humans, this "context" is always changing as their state of health and their present environment or goals change. Relevant "states" of a person with disability can range from a degree of impairment (e.g., spasticity) to a perception of pain, and such states frequently change over the course of a day (e.g., due to medication). Thus a first goal is ***context-awareness ***, which for an intelligent wearable technology includes estimation of relevant states of the person. For instance, how a certain sensed event is interpreted can be influenced by the current "state" of person (e.g., degree of spasticity, pain), as well the history of past inputs (e.g., medications taken recently).

In response to the second of these, our original work on this project was motivated by the desire to create an intelligent system that was based on the mind-set of the rehabilitation practitioner. This led to the aim of designing a prognosis-prediction system that integrated the stages identified in clinical practise guidelines [5], a dynamic process that includes ***diagnosis ***(based on factual and context data), ***prognosis ***(prediction of outcomes, based on certain assumptions), a "clinical algorithm" of ***interventions ***(inputs to the human system), allocation of human ***resources ***(e.g., practitioner time), and ***outcomes ***measurement. While we started from the perspective of planning to use consensus expert experience to build models, a key trend in clinical rehabilitation has been a focus on evidence-based practice [2,5,6]. Also, we noted that the common goal of optimizing therapeutic interventions (e.g., movement therapy) over the continuum of care [6,7] bears striking similarity to classic engineering optimization problem [3].

The above concepts provided the core motivation for our Intelligent Telerehabilitation Assistant (ITA) project [1,3,8,9]. There are two core parts to our vision for mobile ITA technology [1]: **i) **a user-customized interface that supports multimedia teleconferencing and wireless communication, and collection of sensor-and user-based information that can be used to determine events; and **ii) **embedded intelligent "soft" computing, based on event-driven expert system modules. This paper addresses a part of the latter, which to us appears to be the greater challenge. Given this focus, perhaps a better term than ITA, at least for mobile applications, would be a wearable intelligent assistant/agent (WIA). Use of WIA emphasizes the need for context-awareness and prognosis prediction to a greater degree, with the focus on the person rather than on the connection. Aims of a WIA include: i) providing data within an ecologically valid setting, ii) improving timely assessment of health status, iii) identifying and predicting client outcomes (a running prognosis); and iv) assisting with intervention strategies.

Notice the inclusion of both "assistant" and "agent" for a WIA. The former is motivated by the disability community, and the latter by the intelligent systems community. An ***intelligent assistant ***is an assistive technology that directly interacts with and supports the user-client by providing strategic assistance (e.g., with completion of a certain task; providing reminders related to a certain assessment or therapeutic protocol; using performance monitoring to change settings during a therapeutic task). In contrast, an ***intelligent agent ***recognizes events and/or senses data on the user's behalf, and once triggered (normally by using a previously designed rule database), can perform certain actions (e.g., process and manage data, prompt a session between the client and a remote site, negotiate with other agents) while requiring minimal attentional resources by the user. We view ITAs and WIAs as falling into two categories [3]:

• Task-based, assistive modules that facilitate ease of use and implementation of evaluative and therapeutic protocols, and

• Decision-support modules that assist practitioners and consumers with outcomes assessment and with optimizing the rehab intervention strategy.

The present contribution can be viewed as an encapsulated, distributed intelligent processor that is used by a WIA, or more specifically as a resource for a WIA.

Importantly, it is designed in two stages. In the development stage, the designer possesses a suite of tools for creating the model. This model includes identification of:

• ***input ***events and facts,

• the bio-***states ***of interest that are expected to change over time (and whose estimation provides context-awareness),

• performance ***outputs ***to be predicted by the model (and in some cases can be compared to sampled measures), and

• desired ***outcomes ***(optional capability).

All of these are represented as signals, and furthermore signals that change over time. Indeed, the aim of clinical rehabilitation is to cause change that is over-and-above spontaneous healing bioprocesses [3], and to study such processes one must also model intrinsic healing mechanisms. Thus what is needed is a dynamic model that captures change, and can furthermore predict future change (make a "prognosis") if assumptions are made on future inputs (e.g., a "clinical algorithm" of interventions is implemented). The need to model change in states such as "impairment" implies a model that includes differential equations, and the desire to "remodel" the system suggests adaptive control mechanisms. Yet the likely designer of the system is one with experience and knowledge of available evidence, i.e. a practitioner or a clinical researcher. This makes a strong case for using ***rule-based fuzzy inference ***, which is well-known for its ability to both capture expert reasoning and provide robust system performance [10,11]. It also suggests that any model development environment must have carefully-designed graphical user interface (GUI) windows that can help guide the designer through the process of defining linguistically-meaningful signals (inputs, states, outputs, outcomes) and using rules to establish how changes in states will happen in response to input events and current states. More broadly, it can be viewed as a bio-modelling tool for uses rules to generate nonlinear differential equations that can be used by stakeholders ranging from telepractitioners to basic scientists who are addressing healing and remodelling bioprocesses.

When formulated in this way, the structure bears direct similarity to the classic state and output vector equations of systems and control theory, only with the nonlinear state equations developed by fully linguistic and interactive procedures of a rule-based fuzzy inference system (FIS). In our case the equations are implemented via dynamic connectionist neural network (CNN) connections. We thus use "rules" as the bridge between human reasoning and the mathematical model [8-11]. Note that crisp logic can be viewed as a special case of fuzzy logic [11].

Such neuro-fuzzy approaches fall under the umbrella of "soft computing" technologies [10,11], but the approach described here appears to be unique in its focus associating rules with changes in state and thus nonlinear differential state equations created in a linguistic space. Such soft computing approaches have the dual advantages of a structure that can enable robust model behaviour (if designed well) that has made fuzzy controllers such an economic success story, plus use of a intelligent systems architecture that should make it interface well with WIAs decision-making modules. We have coined our general design system SoftBioME (Soft Bio-Modeling Environment, pronounced "soft-by-ohm").

Once designed and customized for a client, in the embedded "run" mode, the model must receive inputs (sensor-events, user-events) as a function of time. The job of the model is then to produce ongoing ***state estimation ***(for context-awareness) and ***useful outputs ***. There are three types of useful outputs: i) performance predictions (e.g., for comparison to actual performance, when measured); ii) specific actions that are a function of states and inputs (e.g., prompting/informing/reminding a client); iii) other value-added decision-support signals for a WIA. Note that it also allows "what if" use by the WIA or a user: it will predict future states, output and outcomes if assumptions are made on future input events.

Developed within the Microsoft .Net Framework using mostly C# code, the "run mode" code is designed to run on any Windows-base system ranging from desktop to PDA. It uses an object-oriented structure, it's support for XML should make it easy to interface with other modules or the web. However, when used in designer mode, it requires a monitor that is large enough to display interface window sizes that are normally intended for desktop/laptops.

## Methods

**Figure 1:**
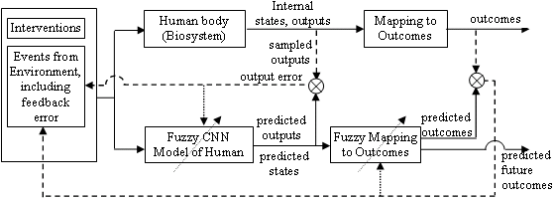
**Structural relation between the model and the real human system. **The intervention plan drives both the real system and fuzzy model, with the sampled (measured) output signals feedback back as an error event signal, and outcome error signals available to mildly tune the adaptive state estimators and output and outcome predictors. Targeted parameters can include input or output mappings or rule weights. When used in a simulation mode, the model can be used to predict the consequences of alternative treatment/intervention plans, and thus help the user optimize the intervention strategy. CNN: connectionist neural network. Dashed line: Sampling. Dotted line: future adaptive CNN work.

The fuzzy system is implemented by a dynamic recurrent neural network that is composed of four layers of CNNs (Figure 1): input, rule-state, output and outcome. Collectively, it is defined by its structure, signals, and parameters (e.g., membership function describing parameters, weights, time constants). We define four roles for users, listed by level of security access:

• User-designers, who have access to all aspects of model creation and implementation, including defining and adding signals, rules and parameters.

• User-analysts, who have access to specifying inputs, to all graphics capabilities, and to using tools such as sensitivity analysis on any internal signals or parameters, but cannot add rules or permanently change parameters.

• User-practitioners, who have access to specifying inputs and storing "what-if" and sensitivity-analysis simulations, as well as full desktop graphics features.

• User-clients, who are often also patients, and have a simpler interface intended for a PDA that can specify inputs, receive outputs, and can obtain current state and output information and summary predictive information.

A given user may participate in (and thus have access to) multiple roles. For instance, an informed and highly engaged patient-client who is active in self-care may normally function in the role of user-client, but can log in to a desktop version where they have "user-practitioner" or "user-analyst" access. Similarly, an experienced practitioner may normally function in the role of user-practitioner, but periodically login as user-analyst and on occasion as user-designer so as to add a new rule or change a membership function or gain. The remainder of this section targets the capabilities of the system from the perspective of the user-designer.

Early versions of this model have been presented as conference papers [8,9]. In the process of using this model for research and for homework projects in rehabilitation courses, it became clear that there was a need to add a number of features:

i) to more fully delineate between and support key dynamic processes associated with different forms of inputs;

ii) to set up a rule structure that enables parametric time constant changes;

iii) to define and implement homeostatic states; and

iv) to support advanced sensitivity and optimization tools.

This paper presents this refined structure, with a special focus on two areas of special interest for WIAs: state estimation for context-awareness and outputs/outcomes prediction for prognosis updating. The model of Figure 1 is presented in a right-to-left progression, since a user-designer normally starts by identifying desired outcomes and outputs.

### Outcomes Layer: Predicting Client Outcomes

Outcomes are defined as scalar signals that relate to what in engineering optimization are called performance sub-criteria or cost functions, and can be a function of fuzzy states and outputs (and if desired, also inputs). Outcomes are thus what a "clinical algorithm" seeks to maximize or minimize. Examples of rehabilitative outcomes are numerical representations of terms such as impairment, disability, independence, quality of life, satisfaction, and cost. An outcome is calculated as a weighted sum or a weighted sum of squares of dimensionless state signals (*X *) and state expressions (e.g., result of "state is low", called *M *_*x *_), and output (*Y *) signals. Weights are selected by the user-designer from a menu table.

### Output Layer: Converging Signals to Predict Performance

As with a conventional control system, outputs are linguistic variables that are function of states and inputs, and change value dynamically only as states and/or inputs change. A given output typically falls into one of three categories:

i) performs an action (e.g., prompt WIA or user-client, initiate communication, store data in an electronic record),

ii) predicts a performance metric, preferably of a quantity that can be sampled on occasion (e.g., a measure such as a clinical scale or biomechanical metric), or

iii) provide targeted decision-support information of use to the user.

**Figure 2:**
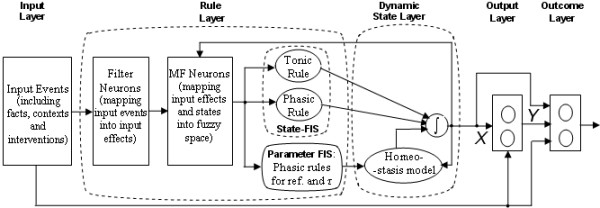
**Layer structure of the model. **Most of the neurons in the input layer detect the occurrence of events and mapping the events into fuzzy variables. Others are pre-processing neurons for certain types of inputs, such as performing as pharmacokinetic models to map the dose and/or regimen of one kind of medication into the effective concentration, or integration neurons to calculate the accumulative effect of interventions. For each state, there are generally five nuclei in the rule-state layer. The outputs of tonic rules nuclei determine the absolute value of the state, and the phasic rules nuclei brings the instant change to the state. (Specially, the nuclei connect the fact/context and the states as tonic rules and phasic rules, with neuronal leaky integrators defined by a time constant to describe how fast the caused change in states reaches its result value.) One nuclei functions as homeostasis mechanism, whose reference is given by the output of phasic rule for reference nuclei (see also Figure 3). The last nuclei works as a math model to relate the Type B interventions and the change of the state. The output of the integration neuron in the rule-state layer is the state X, which then along with inputs are mapped into output Y. The outcome J is a function of all inputs, states, and outputs.

The output of the *ith *output-neuron in this layer, *y *_*i *_, is a function of the states of the rules and the input events (see figure 2).

*y *_*i *_= *f *(*X *, *M *_*U *_, *M *_*X *_)     (1)

where *X *are state signals, *M *_*U *_are the values of membership-neurons based on fuzzy input-MF mapping, *M *_*X *_are membership-neuron values for fuzzy state-MF mapping.

The function *f *can be a Sujeno fuzzy inference system [11] or a weighted sum, selected by the user-designer. Depending on the application and the user-designer's intent, the output can be treated as a fuzzy or crisp value.

When output predictions are of measures that can be experimentally sampled, the user can determine an error signal. Such sampled errors can be viewed as a form of corrective "context" input that can be used to help tune future states and outputs.

### Rules and State Layer: Nuclei Generating Differential Equations

States in this model are fuzzy linguistic variables that are dynamic estimators of physical, physiological and/or psychological states of the human body, of body impairments and of risks. They are modelled as dimensionless signals that can change value as a function of time, based on rules designed within a fuzzy expert system that serve to set up the dynamic state equations that are implemented as a CNN. The rule-state layer consists of a nuclei (cluster of neurons) for each state (see Figure 2), with each nuclei essentially implementing a nonlinear differential equation for that state that can also include recurrent connections from all states, including self-connections.

The fuzzy inference ("expert") system (FIS) consists of a left-half side (LHS, also called "if" or "antecedent" side) and a right-half side (RHS, also called "then" or "consequence" side). As is conventional for a FIS [11], each linguistic state variable has one or more fuzzy sets (represented by a linguistic "value") that are characterized by associated membership functions (MFs) over the variable's Universe of Discourse, such that a state membership value (*M *_*X *_) represents the "degree of membership" of the state variable *x *in a fuzzy set (linguistic value), or the "degree of truth" that "*x *is value." The result is a number on the interval <0,1>, where "1.0" is full membership. Each rule may include any combination of state memberships (*M *_*x *_) and input memberships (*M *_*u *_) on the LHS, and must include a state membership value calculation (*M *_*x *_) on the RHS that indicates how the state would change. Classic fuzzy operations (AND, OR, NOT) and hedges (VERY, MORE-OR-LESS) are supported, and easily added to rules through an interactive GUI. The end result is that the LHS provides a "strength" of firing for the state-change operation(s) described on the RHS.

Of note is that while the logic of the FIS is a function of the states *x *and input effects u * occurring at the same time iteration and thus is a nonlinear static mapping, there are dynamic operations both after and often prior to this FIS operation. The form of the RHS determines the manner of desired change in the state. Rule consequents that target the absolute value of the affected state are implemented by tonic-neurons, while rule consequences that target a relative positive or negative change in state are implemented by phasic-neurons. The dynamic effect of the FES on a state is determined by which of two classes the state is associated with, as is now discussed.

#### 1) Group I: Conventional Fuzzy States

Conventional states change over time based on one or more rules. For one state *x *_s_, normally the spontaneous recovery procedure is:



where *x *_*r *_is the new drive, based on weighted consideration of the current strength of rules associated for a given state, as implemented by the state's nuclei. The time constant τ represents first-order dynamics.

There is also a FIS associated with ***dynamically changing the time constant ***of the rules as a function of states and inputs on the LHS. This is a feature that needn't be part of the user-designer's strategy, but is really quite a powerful addition since it makes available a range of possibilities for state transition dynamics. For instance, the popular Michaelis-Menten kinetics [12] and various cell growth laws [13] can be mathematically viewed as state-dependent variable time constants (inverse of rate constants) that represent special cases of the menu of possibilities.

While all linguistic states can be treated as dimensionless fuzzy signals with first-order dynamics that use a variable time constant that can also be set by a fuzzy rule, based our experiences and those of students using versions of the model in courses, there is also a need for another class – homeostatic states, which are described next. Examples of states that are inherently non-homeostatic are pain, skill, balance and risks.

#### 2) Group II: Homeostatic fuzzy states

While conventional fuzzy signals can always be used when evidence and/or expertise is available, our experience has been that many states are not well captured by first order dynamics because they are part of more involved internal body processes. Thus many physiologic and functional states of the body, including both measurable signals and linguistic variables, are part of inherent homeostatic systems. For instance, physiologic measures ranging from body core temperature to heart rate are regulated, and after a tissue injury there are intrinsic healing mechanisms that aim to minimize the degree of impairment. All these states are controlled by a negative feedback loop. Thus this class of states can include nearly all physical and physiological signals, from blood pressure to muscle strength.

**Figure 3:**
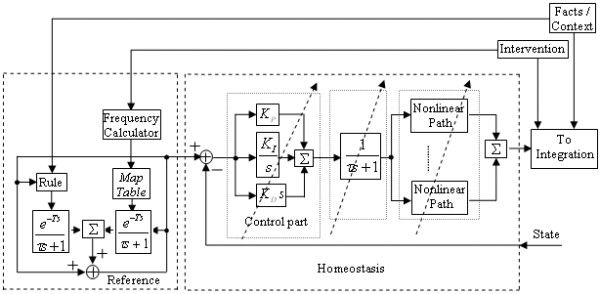
**The structure of nuclei for reference and homeostasis. **A fact event can changes the reference via its own FIS (Rule Type A), and the change will be added to the reference through a first order system with a certain time of delay. When a context event happens, it will affect the reference in the same way as fact events. When there is an intervention, its frequency at the point will be calculated based on the history by a frequency calculator. A user-defined mapping function will then be applied to calculate the change. The mapping function maps the frequency and intensity of the intervention and the initial reference value into the result change. Then the change will be added to the reference through a first order system with a certain time of delay. The mapping function is defined by the user as two tables. If the frequency or the intensity is not in the table, the result change will be calculated by interpolation. All the result changes on the reference of one state caused by different inputs will be summed together by fuzzy OR operation, and then applied to the reference value. Users are encouraged to change references slowly and conservatively. The homeostasis nuclei sense the state value and compare it to the reference. Its output is sent to the integration neuron in the rule-state layer. In homeostasis nuclei, each path in control part and nonlinear paths and the feedback path can be turned on/off by the user. The fuzzy OR operation is used to assure the stability.

In determining the modelling strategy for such states, it is important to recognize that the user-designer's experience is typically with the closed-loop system, with no real knowledge of open-loop dynamics. Thus a challenge is to extract closed-loop knowledge of temporal dynamics and reference state to implement elements within the framework of a "plant" and "controller," and a reference ("set-point") input that itself can change through an intrinsic remodelling process. The current algorithm for how the homeostatic states maintain their equilibrium under the effect of different kinds of inputs is demonstrated in Figure 3, and includes a PID (proportional-integral-derivative) controller to represent the real capabilities of neurons for neural differentiation (e.g., primary muscle affects) and neural integration (e.g., brain stem interneurons). For any homeostatic state, there are two values in this model: the reference and the actual dynamic state.

The reference is the value that represents the homeostatic "ideal" for the human body. If, for any reason, the actual state value deviates from the reference, the controlling organs such as the nervous system and glands will, by sending control signals, try to drive the actual state value toward the reference. Homeostatic references may change under the effect of both internal and external factors. Internal factors include developmental growth and the aging process. External factors include trauma causing impairment and/or lifestyle changes. When intrinsic homeostatic recovery processes are not successful or lifestyle changes are sustained, certain states may gradually adapt to a new reference.

Often D-action is zero unless there is an initial sharp response to a sudden input effect. In such a case an initial closed-loop time constant provided by the user-designer relates primarily to P-action. There is often then a slower drift toward homeostasis and/or remodelling, which can be used to estimate I-action and slow (near-permanent) transitions in reference.

As seen in Figure 3 this model contains two parts: the subsystem for the actual state value and the subsystem for the reference, both of which work as a feedback control system. In the former, the human body senses the actual state value and compares it to the reference. The error between them is the input to the control part, which represents the neural system and glands. The fuzzy OR operation is used as summation because of the physical limitation of the control signal. After the first order plant, the model supports nonlinear paths to capture plant-based nonlinear characters such as time delay or saturation (e.g., a fact event of injury may cut off or activate some specific nonlinear rehabilitation pathway); at present this has not yet been used, and research on optimizing the homeostatic feedback process continues.

To summarize, users specifying "homeostatic states" need only provide general closed-loop temporal and steady-state behavior, and a reasonable but conservative homeostatic regulator is automatically implemented.

#### Pragmatic Consideration: Separate Use of the FIS for Other WIA Modules

While the rule structure in the model is set up for addressing changes in dynamic states within a FIS framework, static rules and crisp logic are just special cases where the post-FIS time constant is zero and MF's have a hard boundary, respectively. Thus a WIA could also use this model, for instance, to create a separate FIS module that uses simpler, conventional real-time crisp logic, where states-to-output mapping is trivial (states equal outputs) or serves to perform aggregation/defuzification.

### Input Layer: Classification and Implementation

Operations within the input layer depend on the type, with inputs classified into facts, contexts, and interventions. This layer can be viewed as a collector and pre-conditioner of inputs, designed to help map them to fuzzy "input effects" that are used in the rules that determine the state equations. Options include pre-filters such as physical models (e.g., a pharmokinetic model for Intervention Type-A (medications)) that are implemented prior to mapping to the fuzzy linguistic world via MF's that are associated with the input's fuzzy values.

In general, MF's are defined by two parameters that define either Gaussian and boundary (sigmoidal) shapes (states also have a monotone option). While these shapes provide continuous derivatives (good for many CNN algorithms), the boundary option does support the special case of a hard (crisp) boundary.

#### Facts

FIS systems often call their inputs "facts." As used here, facts are linguistic variables with a universe of discourse (range) that can be turned on but not normally turned off. In rehabilitation and sport medicine, these are often associated with the patient healthcare record, and include demographic information (e.g., age, gender, education level) and the occurrence of some diseases and diagnosis information (e.g., severity and localization of an event such as a stroke; co-morbidities). Each fact variable has at least one associated fuzzy linguistic value (each with an associated MF on <0,1>).

The relations between inputs such as facts and states are represented within fuzzy rules in the FIS, as describe previously. However, before a fact-event is used in the FIS, it is first mapped within the early part of rule-state nuclei into a "fact-effect" by a first-order time constant selected by the rule-designer (with default value of zero). Since a fact-event provides a step change (and thus a fact-effect a first-order step response), if one fact-effect was the only input on the LHS (i.e., a "fact-effect is value" yielding a *M *_*u *_number), the overall state change would be up to a second-order (overdamped) step response (one time constant before the FIS calculation that maps the "input event" to an "input effect" and is associated with the rule, and one after that is associated with the state). Individual facts thus can trigger rules to fire and cause changes in values of certain states, and possibly changes in the state's time constant and/or the reference value if the state is a homeostatic state (see Figure 3).

#### Context Inputs

Contexts are inputs that can be turned on or off, and make event-based "context awareness" available to the FIS for state estimation [1]. Normally they relate to external environmental events that can have an impact on the state of the person, but there are no limitations placed on context inputs. For instance, in stroke rehabilitation the clinical prognosis is a function of factors such as the ongoing degree of supports (e.g., social, caregiver, family), the clients diet and other nutritional concerns, the location and type of rehabilitation that is available, the client's normal daily or weekly life events, variation in their degree of motivation or ability to achieve lifestyle modifications, assistive technologies that are available to support independence, and so on. All can be viewed as context inputs, as can some interventions as long as the user-designer doesn't desire to use the types of more sophisticated mappings discussed in the next parts of this section.

Context inputs are important for WIA's, and are often used in tandem with state estimates for WIA decision-making. To some extent, they can be viewed as "temporary facts" that help sculpt rules, often weakly but occasionally strongly. Often they help add robustness and integrated realism to the rules and thus state estimation.

The form of the relations between contexts and states are the same as that between facts and states, except that the effect is a pulse (rather than step) response. The change of the status of one context (from off to on, or from on to off) is treated as a context event, which in turn may cause rules to fire differently.

#### Interventions

Interventions are a purposeful procedures and techniques aimed at producing changes in the condition consistent with the diagnosis and prognosis. Interventions may occur regularly or irregularly. Relative to the temporal dynamics of adaptive change, interventions can usually be viewed as impulses to the system. While interventions can always be treated as context input events of short duration, it is useful to develop evidence-based customized approaches for dealing with certain classes of interventions that are common in rehabilitation.

Although one individual intervention often only brings an "impulse response" change to state values because of length of time required for adaptive remodeling, available evidence or professional expertise may be available that indicates that a series of one type of intervention – a treatment "dosing" plan such as three sessions per week – may gradually change the reference value since the human body is an adaptive system. Often scientific studies provide evidence of remodelling based on a global dosing algorithm that is maintained for weeks or months. Adaptation thus can be due to the integration of the responses of the body to each intervention, and to slower intrinsic changes in homeostatic reference values. Based on the mathematics used to mapping intervention inputs to the effect on states, interventions are currently classified into three types.

#### 1) Type A: Medication

This type of intervention supports both oral and injected medications or special dietary measures. In order to describe the effect of a medication, pharmacokinetics (the study of the bodily absorption, distribution, metabolism, and excretion of drugs) and pharmacodynamics (the study of the time course of pharmacological effects of drugs) are included in this conventional (non-fuzzy) model that is implemented within the input layer. The common methods in pharmacokinetics, which are consequently used in this model, are compartment model and Michaelis-Menten kinetics [12]. There are several different mechanism-based pharmacodynamics models [14], each applicable in certain conditions. Essentially, pharmacodynamics is the mapping between the concentration of certain drug and its "effect" on the state. Therefore, fuzzy logic as a very powerful non-linear mapping tool is adopted to implement the pharmacodynamics in this model.

As shown in Figure 2, when there is an event of medication, at first it is mapped into a time series, which represents the concentration of that medication in the blood or other destination spots, through a pharmacokinetics model. If it is an oral medication, a compartment model with two compartments (gut and blood) and Michaelis-Menten (M-M) kinetics are used. The former describes how fast the drug transfers from gut to blood, and the latter calculates the consuming velocity of that drug in blood. Assuming the mass and concentration in the gut are *m *_*1 *_and *C *_*1 *_and in the blood are *m *_*2 *_and *C *_*2 *_, the diffusion constant between the gut and blood is *K *, and the constants of M-M kinetics are *V *_*max *_and *K *_*m *_, the equations are:





If injected, only the M-M kinetics equation is applied. As part of a collaborative project with a post-doctoral fellow (Nicole Sirota, D.O.), estimated values have been tabulated for over 40 medications commonly administered by rehabilitation physicians. The concentration is then an input to a Tsukamoto fuzzy inference system [11,15] to determine the dynamic effect on target states, for use in the rule-state layer.

#### 2) Effective Pulse Energy

Possible inputs of Intervention Type B include exercise, language therapy, recreation therapy, etc. In this type of intervention, a patient and/or provider provides inputs of magnitude and duration that have associated "energy" that is partially or fully "consumed" – the "effective" input. If subsequent changes in the affected state exhibit temporal dynamics that are long in relation to the time duration of the intervention, the input can be viewed as an impulse with an effective impulse energy; otherwise it is a pulse with a changing "effective" magnitude over its duration. In either case, how much energy is consumed in one intervention relates to whether the pulse energy becomes greater than an accumulation threshold energy, after which it triggers a first-order history-dependent recovery/refractory/fatigue variable that subtracts from the input until full effectiveness is gradually restored. Additionally, if another intervention event of the same type happens during the period of time before full recovery, the effectiveness of that event on states will depreciate. This type of intervention is thus mapped to an input effect that is then used to determine its effect on changes in the affected states. Research in this area continues, and details are not provided here.

#### 3) Anticipated Intervention Types C, etc

It is anticipated that there may be dynamic effects of other interventions not yet modelled, which may be defined by users if evidence suggests dynamic processes (e.g., physical lumped-parameter or compartmental models) prior to mapping for use of fuzzy inference capabilities (e.g., functional electrical stimulation).

## Results

### Example Model #1: State Estimator and Output Predictor for Neurorehab Using Medication & Activity Interventions

This first example demonstrates the model's use in providing ongoing context awareness of a person's state, which is a critical need for future WIAs. A secondary purpose is to predict performance outputs and outcomes prognosis. There are two steps to the interactive design process: setting up the model, and running simulations.

Table 1 describes the inputs, states, outputs and outcomes for a hypothetical client, defined by a problem statement. Design of the system usually proceeds with a right-to-left flow, starting by identifying desired outcomes and performance outputs, and then determining the internal states that ideally would be estimated to determine these measures. However, for the type of context-awareness needed by WIA's, the WIA user-designer may have a need for certain specific state estimates, and there is no requirement that every state map to an output or outcome.

**Table 1:** Signals for Example #1.

Female client with stroke-induced disability a large-scale model with 16 types of potential input events, 12 states to estimate, 5 outputs, and 3 outcomes.			
*Problem statement: *An older woman presents with stroke-induced disability (4 months post-stroke) that includes mild functional limitations to gait and posture, and significant impairment of the right arm and hand and of speech production. She also presents with mild osteoarthritis that affects her hips and knees. Released from outpatient care and living alone, her current "prescriptions" include three types of medication doses (for general joint and skeletal health, for pain from arthritis, and for spasticity), and three types of activities suggested by her former therapist (walking/cycling, hand operation, and oral communication). She also has two important weekly events: a visit most Sundays from her daughter (who is a nurse), and a visit most Tuesday's to the local community center (transportation is provided). She regularly uses a PDA-cellphone and a desktop computer (both set up by her other daughter who is an engineer, but lives in another state), and prefers to use an IP videoconferencing package to tele-visit with either of her daughters. Thus she is a good candidate for an assistive WIA.			

*Inputs (and MF example) *	*States (and MF example) *	*Outputs *	*Outcomes *

Facts: - Age (is old) - Initial Stroke (is severe) - Osteoarthritis (is mild) Contexts: - VisitDaughter (is full) - VisitCommCenter (is full) - LocationByGPS (is outside) - TeleVisitDaught (is active) - TimeOfDay (is morning) - NovelEvent (is negative) Interventions (Meds or Activity) - PillsOsteo (is right-dose) - PillsPain (is high-dose; conc) - PillsSpast (is 2-pills; conc) - Walking (is good) - Cycling (is good-quality) - Speech (is good-duration) - Keyboard (is good-session)	Degree of Impairment: - Gait (is faster) - Balance (is better) - RightArm (is worse) - RightHand (is better) - Speech (is improved) Physiologic: - RestingHR (is higher) - RestingBP (is higher) - BoneJointHealth (is low ) Other ("Degree of ..."): - Pain (is high) - RiskFalling (is high) - Motivation (is high) - SleepAtNight (is restful)	Communication [***Φ ***(Speech, Pain)] HandROM [***Φ ***(Hand)] FIM [***Φ ***(Arm, Hand, Balance, Speech, Pain)] RiskFracture [***Φ ***(BJ-Health, Risk-Falling)] Adherence [***Φ ***(Motivation, Pain, Sleep)]	GenHealth [***Φ ***(all impairment \physiologic states)] Participation [***Φ ***(Communication., Gait] QualityLife [***Φ ***(Weekly-Pain, FIM, Speech, Gait, Adherence, Hand-ROM)]

*Notes: *while one MF value is shown for each input or state, typically there are additional ones. Use hedges such as "not" or "very" or "more-or-less" can lower the number of MFs (and thus parameters) associated with a linguistic variable.

Key abbreviations: MF: membership function; GPS: Global Positioning System; HR: heart rate; BP: blood pressure; FIM: Functional Independence Measure [21].

The desired outcomes are in this case to be maximized. Outputs are performance measures that are a function of several states (e.g., FIM score) and/or represent a predicted measurement based on a state (e.g., hand ROM is one measure of hand impairment). Dynamic state behavior is fully dependent on the rules that map current inputs and states (LHS) to changes in states (RHS).

Inputs are mostly pre-determined, based on practical considerations of available data and events that can be sensed or entered by the user. In this case of a WIA application for a "chronic" case that is customized to the specific user-client, it is likely that the user-designer implicitly assumes the effects of fact-events have already played out and can focus on rules involving context-inputs and intervention-inputs. In other scenarios or for more generic population-based rule sets, facts would be a part of rules.

**Figure 4:**
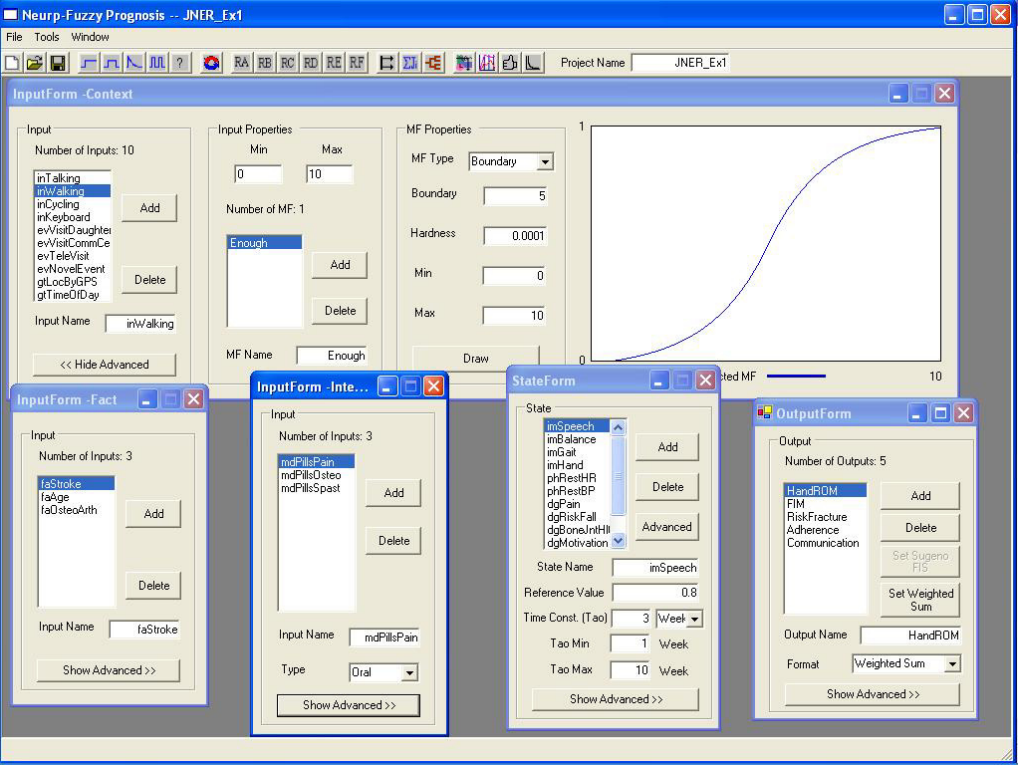
**The interface the inputs, states and outputs in model #1. **There are three facts (bottom left), ten contexts (up), three medications (bottom left two), twelve states (bottom right two) and five outputs (bottom right). User-designer can add or delete inputs, states, outputs or outcomes. For a selected variable, the user-designer is able to set the range (min and max), add/delete membership functions, define the membership functions, and see the graphics of the membership functions. If the variable is a state, the user-designer also has access to the reference, time constant, the negative feedback (on/off), and all of the control parameters.

**Figure 5:**
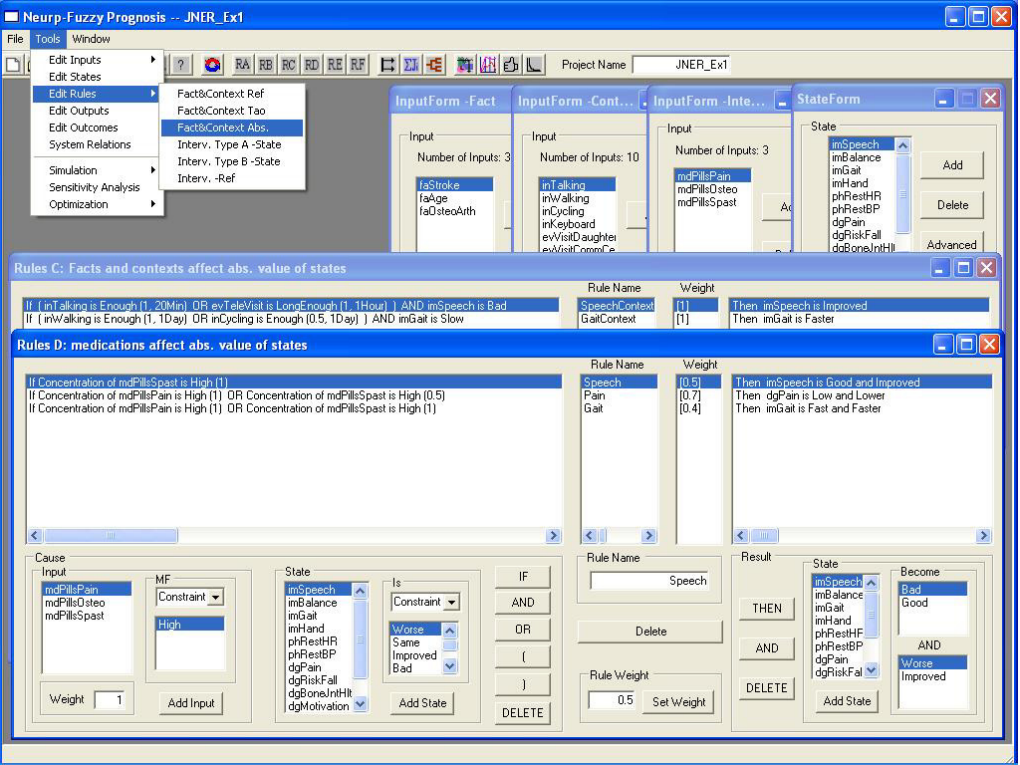
**Type C rules and type D rules in model #1. **There are six types of rules (RA to RF) based on what kind of relation they represent between inputs and states. For example, RC (back window) describes how the facts/contexts change the states' values directly, and RD (front window) defines the relation between medication and affected states. The user-designer can add, delete and change rules, and also change the properties of rules such as rule name and rule weight. When working on "If" side or "Then" side, user-designer can add an element, or delete the right-most element, which is demonstrated helpful for designing rules. In the "If" part, several options are available to help define precise and flexible rules. These operations include: "AND" and "OR" operations, constraints (such as "NOT", "VERY", and "NOT VERY"). There is also a weight for each input element.

To illustrate how rules creation by a user-designer works, a GUI for a representative subset of states from Table 1, and their associated rules, are provided in Figure 4 and Figure 5. Notice that for most rules there is one state-MF on the "then" (right) side, and more than one input-MF and/or state-MF on the "if" (left) side. State-MF's on either side can take express the linguistic expression of a "tonic" neuron "(state is high)" and/or a "phasic" neuron "(state is higher)"; the state "pain," for instance, uses both. Also notice that the rule operates on an "input effect" which is the input mapped through a gain and time constant (see also Figure 2); this allows a rule such as for an impairment state, where changes happen slowly, to integrate context inputs so their effect extends well beyond the time that they are actually on, for that particular rule. Figure 5 also shows that a state, such as pain, can be a function of several rules (e.g., one more related to context inputs, the other medications) that combine through fuzzy operations. Finally, the intrinsic time constant for each state-neuron, differs dramatically between states (e.g., higher value for impairment which changes gradually over weeks versus a measure such as "pain" that can change on the order of hours). These affect logic development. The user-designer needs to understand several features that affect rule design.

**Figure 6:**
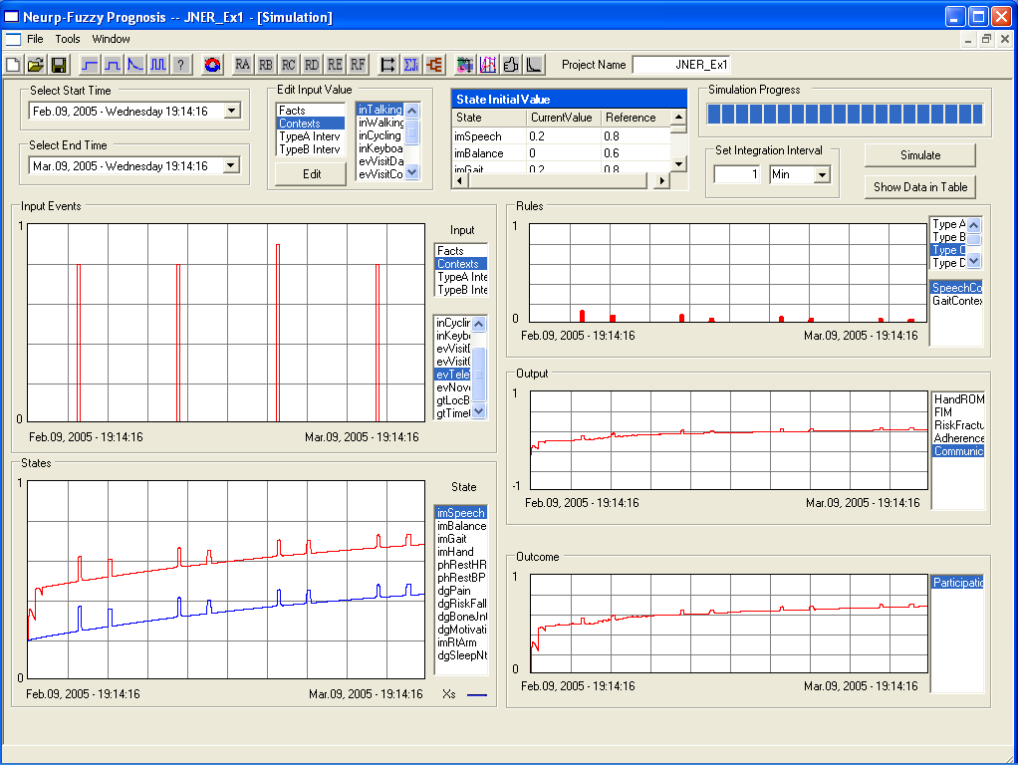
**Example simulation result of model #1. **The simulation period is from Feb. 09 2005 to Mar. 09 2005 (see top left). This figure shows the event train of TeleVisit in the input frame (up left), the status of Speech (bottom left), the rule firing rate of SpeechContext (up right), the output Communication (middle right) and the outcome Participation (bottom right). In the state frame, the blue line is the curve without medication and the red line the curve with medication. Comparing the event train of TeleVisit and the curve of the Speech, we can see clearly the effect of every TeleVisit on the Speech. The other protuberance on the Speech curve is caused by the visit to the local community center on every Tuesday.

An example simulation, with a few weeks of inputs and with states initialized, is presented in Figure 6. Here we focus on the "context" (state estimates) based primarily on "context" inputs, and on a certain slice of time – the "present." Five conceptual points are emphasized here:

Five conceptual points are emphasized here:

1. State change often requires that a combination of input/state conditions occurs, and certain states can change suddenly (e.g., pain) while others only gradually (e.g., impairments, and homeostatic states in general).

2. As with most large-scale nonlinear systems, the "functioning" order of the system, and overall behavior, tends to be only a function of a small subset of the model parameters. As different events fire, different "subsystems" of changing states emerge and the collection of parameters with the highest sensitivity changes. Sensitivity analysis tools, embedded in the model, can be used to gain insight into what parameters matter most at any given time.

3. Many of the expressions on the left-half ("if") side of the rule may be designed to add robustness – they rarely affect the rule, but when they do they effectively drive or shut off firing.

4. While the primary need of WIAs is for context-awareness of state, the fact there is also prediction of the future can potentially be used by a WIA to consider the effects of alternative plans for future events. This may be especially useful for WIA decision-making while functioning in "assist" mode, as there is a fine balance between the benefit of providing a reminder or warning to the client versus the cost of overburdening the client; "what-if" prediction of the future can help in making this decision.

5. While not shown in Table 1 (but evident in Figure 1), of note is that "errors" between a predicted output and measured output (e.g., arm force, hand ROM, FIM and recent pain or adherence can measured by the daughter during weekly visits) can be fed back as context input events that can then be integrated into a rule for a state, helping gradually improve the state estimate.

### Example Model #2: Muscle Force and Joint Strength Changes: Short-Term Fatigue and Long-Term Adaptation

This example illustrates use of the model by a user-designer who has expertise in a certain area plus access to scientific evidence, here demonstrated for muscle strength. One can easily envision an athlete or coach using a WIA to plan and implement an exercise program that has the desired outcome of maximizing muscle strength and tissue hypertrophy over a certain time window, and has estimates of relevant internal states during the process. Similarly, one can envision a musculoskeletal or neuromuscular rehabilitation program that seeks to regain muscle strength or minimize muscle atrophy. In either case, the estimated states and sampled performance output measures can help a WIA to provide a user-client with a suggested input intervention program (e.g., exercise regimen, diet).

This example also exposes another use for the model: by scientists who study bio-change, and in particular who desire to synthesize knowledge of macro-and micro-changes at the organ/tissue and cellular levels, to make model predictions that may be testable, and to bridge human macro-studies with animal micro-studies. Here the onus is on the expert to integrate experience and available evidence. One of us (JMW) has published extensively using neuromusculoskeletal models that include Hill muscle models [16-18]. Hill-based muscle models predict force as a function of muscle activation, length and velocity. In traditional use of such models, parameters are assumed constant for a given simulation. But we know that some parameters do change as a function of activity, and in recent years a growing body of evidence has amassed on how three key parameters [maximum isometric force (Fmax), maximum unloaded velocity (Vmax), a Michaelis-Menten kinetics parameter related to calcium deactivation] change as a function of: **i) **fatigue (a shorter-term reversible change in parameters over a time period of seconds to hours); and **ii) **true muscle adaptation (a more permanent change that occurs over time periods of days to months).

**Figure 7:**
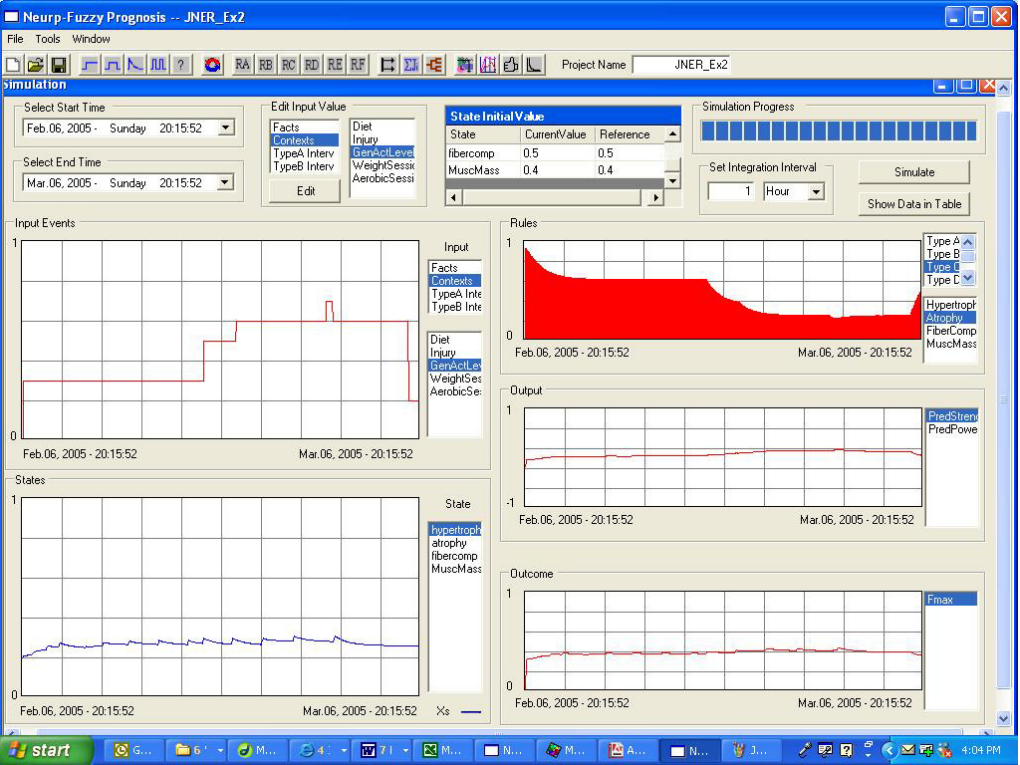
**Example simulation of the "adaptive model" of Table 2. **For this simulation, the following four simple rules were used, one for each state: IF WeightSession is intense & (Diet is good & Hypertrophy is Low) THEN Hypertrophy is high & higher IF (GenActLevel is low & AerobicSess is not intense) or Injury is bad THEN Atrophy is high & higher IF WeightSession is intense & AerobicSess is not intense & FiberComp is low THEN Fibercomp is high & higher IF Hypertrophy is high & (WeightSession is not intense & Diet is not good & AerobicSess not intense THEN MuscMass is high & higher Since only one input, state, rule, etc can be shown in an image (user can easily toggle between them), others are described here. At the start the client has states that reflect a sedentary lifestyle. Inputs reflect that he gradually increases his general activity level (this is the input that happens to be shown), improves his diet, and starts a weight-training program. This continues for three weeks through the end of February, at which time he stops the weight training and starts an aerobic training program. However, on his fourth aerobic event, he gets injured and his activity decreases. The hypertrophy and atrophy states are viewed as bioprocesses that are always somewhat present, and compete with each other. Of the four states, the hypertrophy state is shown (lower left), and we see an initial rise and a subsequent mild effect of each weight training session. After these inputs stop the state falls a bit. The atrophy state follows the shape of the atrophy rule, which is shown (upper right). Notice that with increases in various activities, atrophy rule firing decreases until the injury occurs. The output (predicted strength) is assumed a weighted function of all states, and the "outcome" Fmax (which could have also been viewed as an output) is a weighted function of the predicted strength and some of the states. Both show increases with these lifestyle changes, then the start of a decrease after the injury.

Table 2 focuses on a simple model structure for estimating one of these parameters: Fmax, which also directly correlates to muscle strength and size. It does so on two timeframes, using different models: **i) **for fatigue, the model runs for minutes, with rules structured on the assumption that an exercise "pulse" corresponds to the intensity (percent of maximum) and duration (number of repetitions) of a weight-training "set"; and **ii) **for adaptive change, the model runs for weeks or months, and an exercise "pulse" is the average intensity of a "workout" where a time of an hour is small relative to the dynamics of adaptive tissue change. In both cases these are "converging" models with many inputs; Table 2 keeps these inputs simple. Figure 7 provides an example simulation, here for a client with a sedentary lifestyle who makes a number of positive lifestyle changes but then, after nearly four weeks of training and some improvements in Fmax, gets injured.

**Table 2:** Example of a converging model designed to estimate states, outputs and outcomes.

Inputs	States	Outputs	Outcome
**Fatigue Model:** Facts: • InitFiberComp Context: • Motivation Interventions • SetHiRep60% • SetLowRep80%	CalciumConc P_i_/H^+^-Conc^1^ PossibleReps@80% PossibleReps@60%	PredictedRepsAt60% PredictedMax	Fmax

**Adaptive Model:** Facts: • InitFiberComp Context: • Diet • Injury • GenActivityLevel Interventions • WeightSession • AerobicSession • PillsSteroids	Hypertrophy Atrophy FiberComp MuscMass	PredictedStrength PredictedPower	Fmax Vmax

^1 ^Phosphate and pH concentration, which reflect muscle energetics and transient recovery dynamics; for Fmax, evidence suggests these are are similar in influence (if we added Vmax to the model, available scientific evidence would suggest we separate these states).

## Discussion

This paper develops a novel rule-based neuro-fuzzy dynamic model that is intended to provide continuous state estimation, to predict outputs, and to evaluate the effect of different intervention plans. It enables a user-designer who is an expert in a rehabilitative field, but not necessarily in mathematical modeling, to generate and use causal models that contain underlying nonlinear differential equations implemented with a CNN. To be effective they need to have a solid understanding of the concepts of a time constant, a weight (or gain), how a MF maps a variable, and how negative feedback works; the interactive GUIs can actually be used as a learning tool to help pick up these skills. When creating new inputs and states, default MFs for classic linguistic values such as "high" or "low" are automatically created for the user-designer, using either Gaussian or Boundary fits that are defined by two intuitive parameters – a "middle" and a "shape." These default MFs can be renamed, edited or deleted. The GUI for rule creation, shown in Figure 5, is similar to GUI's for other FIS implementations, only with some added capabilities that are unique to this model. Once the model structure and parameters are set, simulations are managed on a separate GUI that enables the creation of input trains, state initialisation, and other bookkeeping features. In addition to plotting state, output and outcome trajectories, a GUI is available for parameter sensitivity analysis. Once refined to the satisfaction of the user-designer, the model is ready to be used as an embedded application.

The first example shows the application of this neuro-fuzzy modelling framework as a WIA to estimate the current states values and predict key outputs and outcomes. There is no doubt that real-time monitoring of certain key states is valuable for some patients/clients in their daily lives. However, not all of these states can be directly measured by wireless sensors. SoftBioME provides the possibility for an integrated WIA in which wireless sensors measure the measurable events and states and send them to the intelligent agent, while the intelligent agent records these states and estimates un-measurable states, detects the occurrences of pre-defined events (e.g., a state is above or below certain value), and does some pre-processing. This example demonstrated that SoftBioME can provide an estimate of certain states' values at any time. If the user-designer defines crisp MFs and crisp rules, it can also serve as an event-detector. Since the model is designed based on expert knowledge (e.g., how the walking exercise affect the gait) and scientific evidence (e.g., the effect of medications on states), the estimation error shouldn't be beyond expectation. In addition, rules can include error signals on the LHS that are based on any difference between an estimated variable (output, outcome) and periodically measured signals (output, outcome), enabling state estimation to improve over time. Furthermore, by running the simulation repeatedly, an experienced user-designer may adjust the parameters to try to heuristically optimize a customized model before use for real time estimation. The CNN model structure is designed so that in the future a neuro-optimization toolset can be provided to improve the model performance for a certain client, i.e. to "learn" the client's behavior. All of the above promise an accurate-enough estimation for the type of context-awareness that is needed for effective WIAs.

Unlike all the use of macro-states in the first example, the second example contains both macro-states and micro-states. In this example, the macro-states depend on the micro-states and macro evidence from strength training and visa-versa, and that dependence can be described by fuzzy rules. The muscle force model also demonstrated that the model created in SoftBioME can not only estimate states, outputs and outcomes, but also focus on parameters changes. That's because one of the purposes of SoftBioME is to support both signal models and parameter models (e.g., longer-time remodelling models). The parameters in a signal model may simultaneously be the signal in a parameter model, with the two models operating on different time scales (e.g., seconds versus weeks). For example, for some exercise activity performed frequently, Fmax is the signal in the second example and is also a (now adaptive) parameter in the first example (e.g., neuromusculoskeletal model using Hill-based muscles). The ability to work at both signal dimension and parameter enables SoftBioME to deal with a variety of problems in a broad area in rehabilitation.

When designing an intelligent agent through SoftBioME, the most critical thing is to collect expert knowledge and/or scientific evidence. There are several ways to collect expert knowledge, such as Analytical Hierarchy Process (AHP) [19] and Delphi [20]. The latter is often used by doctors and nurses as decision making protocol, which makes it a good choice when creating a rehabilitation model. Published paper and textbooks are the main sources for scientific evidence. Given available expert knowledge and scientific evidence, how to transfer them into membership functions and fuzzy rules is the next challenge. Normally experts will help define MFs and fuzzy rules. If creating an evidence-based model, usually the evidence itself contains the rules implicitly (e,g, abstracts often summarize findings in the form of rules). Sensitivity analysis tools can help refine the MFs and evaluate the importance of rules, which assist the user-designer in improving the model.

Of note is that because of the natural tendency for signal and rule "soft saturation" when using fuzzy models with smooth MFs, the nonlinear differential equations tend to be inherently stable. Most biosignals also have soft saturation at the extremes of their operating range. It is as if the user can think in a more rule-based "linear" and causal manner, but end up with models that, if well designed, are robust over a larger region of the operating state space than for a linearized version of a bio-model.

## Conclusion

A neuro-fuzzy modelling framework (SoftBioME) is developed for estimating changes of states in bio-systems as a function of input event patterns. If carefully designed with sufficient expert knowledge and/or scientific evidence, it can be applied in rehabilitation (e.g., predict intervention outcomes), sport medicine (e.g., evaluate the effect of a training plan), biology (e.g., adaptive changes in muscle), pharmacy (e.g., study the action or effect of drugs), and perhaps other fields whose subject is a dynamic system and adaptive change. It is able to make predictions or real-time estimation. The latter is intended to provide context-awareness of changing states, which is critical for WIA's to be effective.

## Competing interests

The author(s) declare that they have no competing interests.

## Authors' contributions

Both YW and JMW were involved in all parts of this work, with YW responsible for model implementation and most simulations, and JMW responsible for most of the first draft of the manuscript.
